# Population structure and genetic diversity of non-O157 Shiga toxin-producing *Escherichia coli* (STEC) clinical isolates from Michigan

**DOI:** 10.1038/s41598-021-83775-z

**Published:** 2021-02-24

**Authors:** Heather M. Blankenship, Rebekah E. Mosci, Stephen Dietrich, Elizabeth Burgess, Jason Wholehan, Karen McWilliams, Karen Pietrzen, Scott Benko, Ted Gatesy, James. T. Rudrik, Marty Soehnlen, Shannon D. Manning

**Affiliations:** 1 Department of Microbiology and Molecular Genetics, Michigan State University, 1129 Farm Lane, East Lansing, MI 48824 USA; 2 Michigan Department of Health and Human Services, Bureau of Laboratories, Lansing, MI 48906 USA; 3 Michigan Department of Agriculture and Rural Development, East Lansing, MI 48823 USA

**Keywords:** Microbiology, Clinical microbiology, Gastrointestinal diseases, Diseases, Infectious diseases, Bacterial infection

## Abstract

Non-O157 STEC are increasingly linked to foodborne infections, yet little is known about the diversity and molecular epidemiology across locations. Herein, we used whole genome sequencing to examine genetic variation in 894 isolates collected from Michigan patients between 2001 and 2018. In all, 67 serotypes representing 69 multilocus sequence types were identified. Serotype diversity increased from an average of four (2001–2006) to 17 (2008–2018) serotypes per year. The top six serogroups reported nationally caused > 60% of infections in 16 of the 18 years; serogroups O111 and O45 were associated with hospitalization as were age ≥ 65 years, diarrhea with blood and female sex. Phylogenetic analyses of seven multilocus sequence typing (MLST) loci identified three clades as well as evidence of parallel evolution and recombination. Most (95.5%) isolates belonged to one clade, which could be further differentiated into seven subclades comprising isolates with varying virulence gene profiles and serotypes. No association was observed between specific clades and the epidemiological data, suggesting that serogroup- and serotype-specific associations are more important predictors of disease outcomes than lineages defined by MLST. Molecular epidemiological studies of non-O157 STEC are important to enhance understanding of circulating strain distributions and traits, genetic variation, and factors that may impact disease risk and severity.

## Introduction

Shiga toxin-producing *Escherichia coli* (STEC) is a foodborne pathogen estimated to cause ~ 265,000 illnesses in the U.S. each year^1^ with symptoms including diarrhea, hemorrhagic colitis and hemolytic uremic syndrome (HUS)^2^. Most surveillance activities have focused on O157 STEC; however, the incidence of non-O157 STEC has been increasing steadily in the U.S. and surpassed the national incidence of O157 in 2014^3–5^. Six serogroups representing O26, O45, O103, O111, O121, and O145 accounted for 70% and 83% of non-O157 cases reported to the Centers for Disease Control and Prevention (CDC) between 1983–2002^6^ and 2000–2010^3^, respectively. In 2016, the CDC reported that these six non-O157 serogroups remained the most prevalent types nationwide^5^. Nonetheless, few studies have examined the genetic diversity of large non-O157 strain populations comprising multiple serogroups from specific geographic locations.

STEC is classified by the presence of Shiga toxin genes (*stx1* and/or *stx2*) on distinct bacteriophages^7^. Seven *stx2* subtypes have been identified; *stx2* (a-g), *stx2a* and *stx2d* were linked to more severe infections^8,9^, while *stx2e*, *stx2f.*, and *stx2g* were more common in environmental sources and animals^10^. Other virulence genes such as *eae* (intimin) and *ehxA* (enterohemolysin), have been linked to pathogenicity^8,11^. *eae* is found on the locus of enterocyte effacement (LEE) pathogenicity island, which mediates attachment and effacement of intestinal epithelial cells^12^ and *ehxA* is located on distinct plasmids. *ehxA* has been identified in strains from patients, animals, and environmental samples^13,14^. Examining these genes in clinical isolates can help identify combinations linked to severe disease, monitor changes in gene frequencies, and understand STEC evolution.

STEC diagnostics have changed from culture-based to culture independent tests to promote non-O157 detection^15^. The State of Michigan has used a combination of methods since 2001^16^, thereby facilitating the recovery of non-O157 STEC for molecular epidemiological studies. Similar to national trends, the non-O157 STEC incidence has increased in Michigan^17^, though only a subset of these isolates, mainly O157 STEC, has been characterized previously^18,19^. Although many *E. coli* serogroups have been identified, the genetic relatedness of these serogroups and identification of molecular and epidemiological factors associated with infection have not been fully elucidated. Herein, we sought to examine genetic variation and virulence characteristics of 894 clinical non-O157 STEC isolates while identifying risk factors for infection. Examining trends in specific geographic locations can enhance understanding of epidemiology, virulence, and evolution and help guide public health interventions strategies.

## Results

### Case characteristics

A 19-fold increase in the number of non-O157 STEC cases was observed in the 2008–2018 time period relative to the years 2001–2007 (Supplementary Fig. S1). Over the 18-year period, 1,060 non-O157 STEC case reports were recorded in the Michigan Disease Surveillance System (MDSS), the online communicable disease reporting system for notifiable infections. The number of non-O157 isolates recovered for WGS outnumbered the case reports through 2006 when an increasing trend was observed.

The average patient age was 29.0 years (range: 1 day-102 years) over the 18-year period, though the age distribution fluctuated before 2008 (Supplementary Fig. S2). Most cases (45.0%) were between 11–29 years, the predominant age group in 13 of the 18 years examined. The fewest infections were reported in children ≤ 10 and the elderly, while more females than males (p < 0.0001) were affected (Supplementary Table S1). The proportion of females was significantly higher in the later (p < 0.0001) time period and in three of the four age groups:11–29 (n = 219/383;p = 0.005), 30–64 (n = 165/230;p < 0.0001), and ≥ 65 (n = 49/75;p = 0.008). Significantly more infections also occurred in the summer months and among residents living in rural counties, specific regions of Michigan, and counties with high cattle densities.

Among all cases with data available, 29.7% were hospitalized. Females, patients infected during the winter, spring, or fall, and those reporting body aches, diarrhea with blood, and cramping were significantly more likely to be hospitalized in the univariate analysis (Supplementary Table S2). An increased likelihood of hospitalization was also observed with increasing age; elderly patients had the greatest odds of hospitalization relative to children ≤ 10. No association with hospitalization was observed for urban versus rural residence or high cattle density. Multivariate logistic regression confirmed the univariate associations indicating that season, age ≥ 65 years, diarrhea with blood, and female sex were the strongest predictors of hospitalization.

### Serogroup and serotype distributions

In all, the non-O157 STEC infections were caused by 67 different serotypes representing 47 serogroups (Supplementary Table S3). Among these serogroups, 12 (25.5%) comprised isolates with more than one H-antigen, though one predominant H-antigen was observed for each. The 220 serogroup O103 isolates, for instance, mostly had H2 (23.5%) but a subset had H11 (2.5%), H19 (0.1%), and H25 (0.3%). Additionally, 16 non-typeable (NT) isolates possessing nine different H-antigens were recovered.

**Figure 1:**
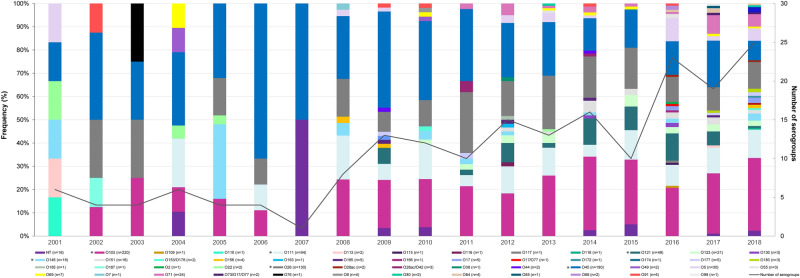
Frequency and number of non-O157 Shiga toxin-producing *Escherichia coli* serogroups reported in Michigan by year. The y-axis on the left represents the frequency of each serogroup by year and is indicated by the different colored bars. The y-axis on the right shows the number of serogroups per year indicated by the black line. The top six serogroups are indicated with an * in the figure legend. *NT* non-typeable.

The serotype distribution was significantly different over the 18-year period (Mantel–Haenszel p = 0.0031). The top six predominant serogroups, or the “big six”^6^, caused > 60% of infections for 16 of the 18 years (n = 699) and were 3.6 times more common than all other serogroups (n = 195; p < 0.0001) (Supplementary Fig. S3). Serogroups O45 and O103 predominated causing an average of 28.6% and 19.2% of infections per year, respectively, followed by O26 (13.7%) and O111 (8.5%) (Fig. 1). While O145 and O121 serogroups caused an average of 3.9% and 3.3% of infections each year, O121 was not detected before 2009. Among all other serogroups, 24 (54.5%) were recovered in more than one year and 20 (45.5%) were recovered from only one patient throughout the study period. Importantly, serogroup diversity increased over time from an average of four serogroups per year from 2001–2007 to 15 from 2008–2018. Serotype diversity also increased from an average of four serotypes in the earlier period to 17 in the latter. No difference in the distribution of H-antigens was observed over the years (Mantel–Haenszel p = 0.539). The H2 (n = 428; 47.9%) and H11 (n = 174; 19.5%) antigens predominated followed by H8 (n = 95; 10/6%) and H9 (n = 30; 3.4%).

### Genetic diversity and recombination

**Figure 2:**
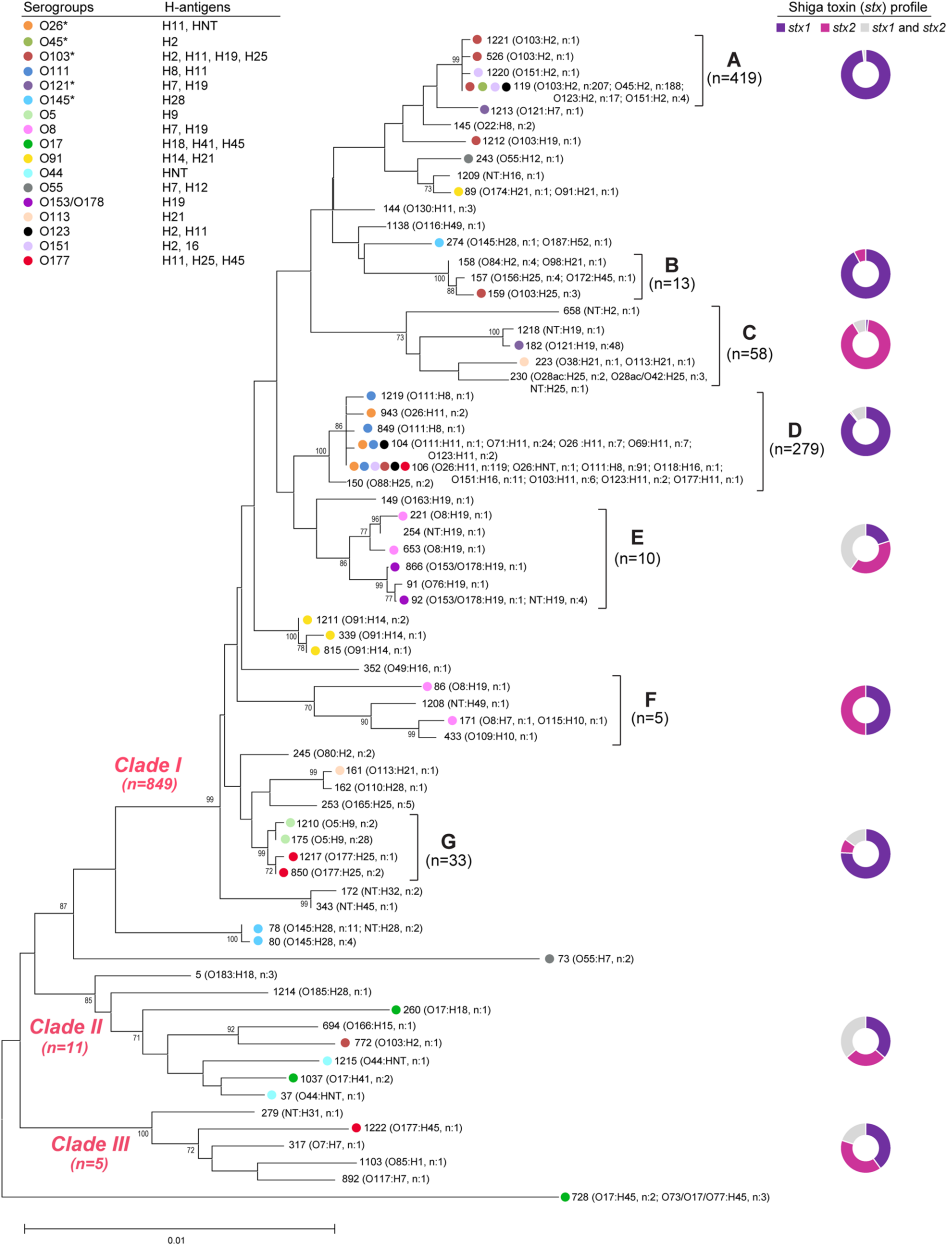
Neighbor-joining phylogeny based on seven multilocus sequence typing loci (3738 bp) among non-O157 Shiga toxin-producing *Escherichia coli* isolates from Michigan, 2001–2018. The nodes at each branch represent the support percentages after bootstrapping (1000 replicates). The Maximum Composite Likelihood method was used to calculate the evolutionary distances (number of base substitutions per site). Three clades were identified that clustered together with > 70% bootstrap support, while seven subclades, A-G, are shown within clade I. Sequence types (STs) are noted at the end of each branch followed by the serogroup and number (n) of isolates per serogroup. The big six serogroups and a set of additional serogroups that were found on multiple branches of the phylogeny are represented by different colored circles. The frequency of Shiga toxin (*stx*) genes per subclade and clade is shown in the pie charts with different colors representing the three toxin gene profiles.

The 889 isolates grouped into 69 STs. Fourteen novel lineages including STs 1208–1215 and 1217, which had new allele combinations, were identified along with five others (STs 2018–2022) containing novel SNPs. ST-119 (n = 416;46.8%) and ST-106 (n = 232;26.1%) predominated followed by STs 182 (n = 48;5.4%), 104 (n = 41;4.6%), and 175 (n = 28;3.2%). The remaining 64 STs comprised < 1.5% of the total with 39 (56.5%) representing only one isolate. Three clades were defined (bootstrap support ≥ 70%) as well as four singletons, or lineages that are not part of a cluster (Fig. 2). Most isolates belonged to clade I, which was divided into seven subclades (A-G) based on bootstrapping and inclusion of ≥ 3 STs. Isolates from subclades A and D predominated overall (n = 698; 78.5%) and in all but one year (Supplementary Fig. S4); these subclades contained 89.9% (n = 626) of the 696 isolates representing big six serogroups.

Multiple serotypes were found on different branches of the phylogeny (Fig. 2). The most diverse lineages were STs 104 and 106 of subclade D and ST-119 of subclade A, which comprised 4–10 serotypes. In several cases, the distribution of serogroups, particularly those representing the top six, was linked to the H-antigen distribution. O103 isolates, for instance, were found across subclades and those with H2, H11, H19, and H25 were on different branches of the phylogeny. Similarly, the 48 O121:H19 isolates belonged to subclade C that was distinct from the O121:H7 isolate (ST-1213) near subclade A, while the three O145:H28 isolates were singletons on two different branches. The remaining top six serogroups were restricted to one subclade. All 188 O45:H2 isolates were in subclade A (ST-119) and the O26 and O111 isolates comprised multiple STs within subclade D. All but one O26 isolate possessed H11, however, the O111 isolates of ST-104 had H11 and those of ST-106 had H8. The distribution of H-antigens was also significantly different across the phylogeny (Mantel–Haenszel p ≤ 0.0001). Isolates from subclade D had four different H-antigens compared to those from subclades A and E, which all possessed H2 or H19, respectively, despite the presence of multiple serogroups and STs (Supplementary Fig. S5). The remaining subclades contained isolates with two to four H-antigens each.

**Figure 3:**
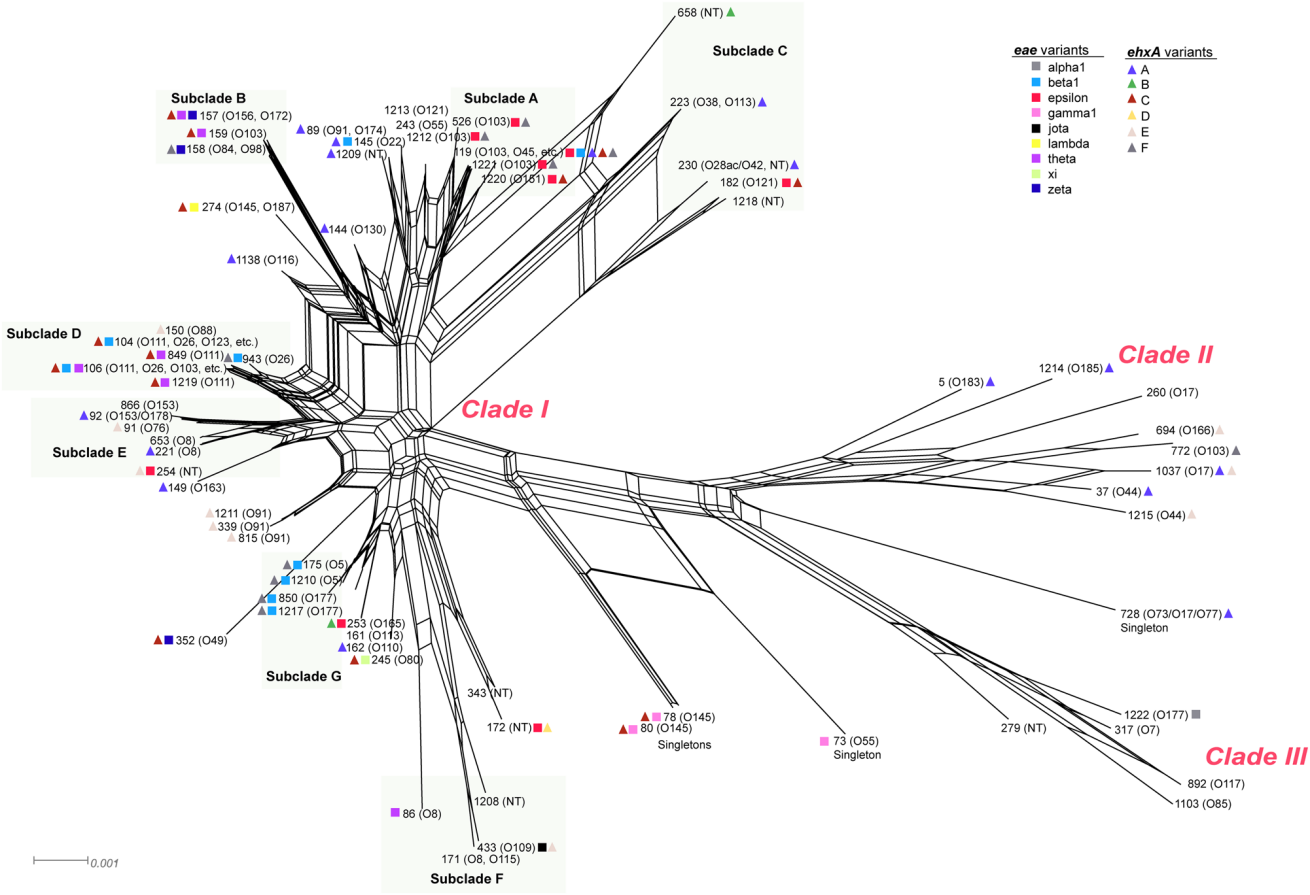
The neighbor-net analysis of 194 parsimonious informative sites among 69 non-O157 Shiga toxin-producing *Escherichia coli* multilocus sequence types (STs). The phylogenetic network was constructed from untransformed distances using Splitstree4. Recombination is indicated as parallelograms and illustrate multiple paths between most STs. The pairwise homoplasy index (PHI), which was used to test for recombination, was significant (p ≤ 0.00001). The network confirms the tree structure of the neighbor-joining phylogeny and clearly delineates the three clades and singleton STs. The five clade I subclades are shaded with light green rectangles. The STs are indicated at the end of specific branches followed by the serogroups represented. Colored squares represent the intimin (*eae*) allele identified, while the colored triangles are the enterohemolysin (*ehxA*) subtypes.

A neighbor-net analysis of all 69 STs detected significant recombination (Fig. 3). Although bootstrap values were low for inclusion of some STs within subclades in the neighbor-joining phylogeny, some STs (e.g., STs 1212 and 149) were more closely linked to specific subclades in the network analysis. The level of recombination for most clade I subclades was extensive, though the STs comprising subclade B were more closely grouped together at the end of longer branches. Given the multiple parallel paths and the significant pairwise homoplasy index (PHI) p ≤ 0.0001, recombination likely contributed to the emergence of STs in clades II and III, which appear to have diversified and are now more distantly related to the clade I lineages.

### Associations with serogroups, serotypes, virulence genes, and lineages

An evaluation of the epidemiological data showed that males were significantly more likely to have infections caused by the big six serogroups (OR 1.5; 95% CI 1.10–2.13) as were cases reporting diarrhea with blood (OR 1.9; 95% CI 1.19–3.08) (Supplementary Table S4). Stratifying by serogroup, O45 infections were associated with bloody diarrhea (OR 1.5; 95% CI 1.01–2.30) and hospitalization (OR 1.8; 95% CI 1.18–2.72), while O111 infections were linked to bloody diarrhea (OR 2.0; 95% CI 1.05–3.66) and cramping (Fisher’s *p* = 0.017) compared to all other serogroups. Patients between 11–29 years were more likely to have O26 (OR 1.5; 95% CI 1.04–2.22) and O45 (OR 1.4; 95% CI 1.00–1.91) infections. Interestingly, O45 and O103 infections were significantly more common in counties with higher (OR 2.3; 95% CI 1.46–3.74) and lower (OR 0.5; 95% CI 0.29–0.70) cattle densities, respectively, when compared to all other serogroups. These relationships were also examined by serotype while considering subclade designations (Supplementary Table S5). Although O45:H2 isolates were restricted to subclade A, no association was observed between subclade A and high cattle density, hospitalization, bloody diarrhea, or specific age groups. The O103:H2 isolates associated with low cattle densities were also restricted to subclade A, which could have canceled out any relationship between cattle density and lineage. Similarly, no association was observed between subclade D containing all 93 O111:H8 isolates and bloody diarrhea or cramping, suggesting that serogroup- and serotype-specific associations are more important than lineage.

### Variation in virulence genes

**Figure 4:**
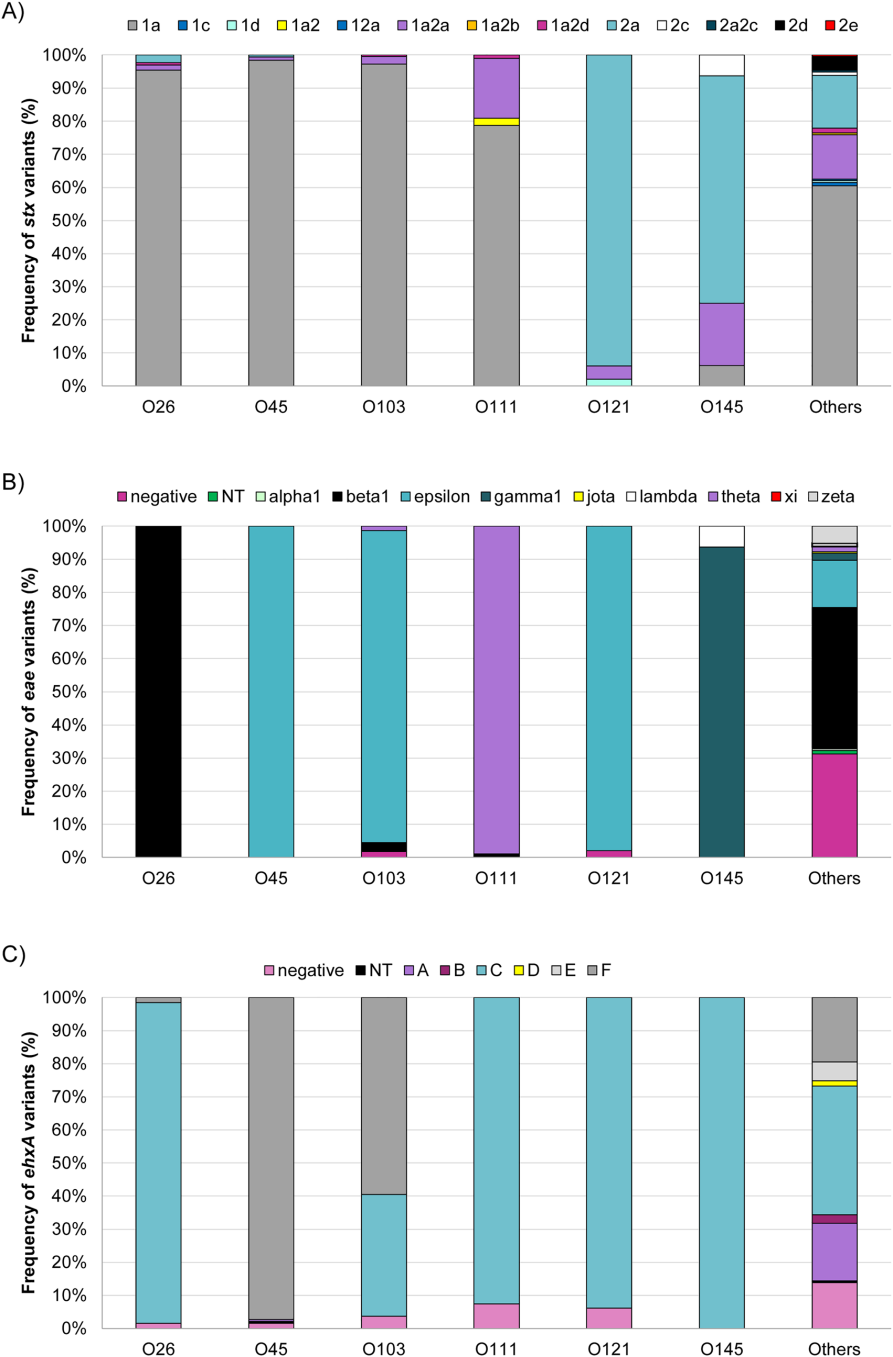
Distribution of gene alleles encoding the (**A**) Shiga toxins (*stx*), (**B)** intimin (*eae*), and (**C**) enterohemolysin (*ehxA*) among the predominant non-O157 Shiga toxin-producing *Escherichia coli* serogroups in Michigan. NT = non-typeable.

In all, 11 gene profiles were identified with most (89.0%) isolates having *eae*, *ehxA* and *stx1* (Table 1); 18 (2.0%) lacked *eae* and *ehxA* and 8.9% lacked *eae* (n = 48) or *ehxA* (n = 32)*. stx1a* (n = 718; 80.3%) predominated, though some *stx1a* isolates also had *stx2a* (n = 57), *stx2b* (n = 1) or *stx2d* (n = 6). Two isolates harbored *stx1c* and *stx1d*. Isolates with only *stx2* had *stx2a* (n = 92), *stx2d* (n = 8), *stx2c* (n = 3), or *stx2e* (n = 1). Stratifying by serogroup showed that *stx1a* predominated in O26 (n = 127; 97.7%), O45 (n = 189; 99.5%), O103 (n = 220; 100.0%) and O111 (n = 94; 100.0%) isolates (Fig. 4A), while *stx2a* predominated in O121 (n = 48; 98.0%) and O145 (n = 14; 87.5%). *stx2c* was found in one O145:H28 and three O177:H25 isolates, whereas *stx2e* was in one O8:H17 isolate. Eight other serogroups harbored *stx2d*.

**Table 1:** Frequency of virulence gene profiles in 894 non-O157 Shiga toxin-producing *Escherichia coli* isolates from Michigan patients, 2001–2018.

Virulence profile	No	(%)
*stx1, ehxA, eae*	676	(75.6)
*stx1, eae*	26	(2.9)
*stx1, ehxA*	10	(1.1)
*stx1*	10	(1.1)
*stx2, ehxA, eae*	73	(8.2)
*stx2, eae*	3	(0.3)
*stx2, ehxA*	21	(2.4)
*stx2*	8	(0.9)
*stx1, stx2, ehxA, eae*	47	(5.3)
*stx1, stx2, eae*	3	(0.3)
*stx1, stx2, ehxA*	17	(1.9)

All O45 and O121 isolates plus most O103 (94.1%) isolates had *eae*_epsilon_ (Fig. 4B), whereas all O26 isolates had *eae*_beta_. The O111 and O145 isolates mostly had *eae*_theta_ (n = 93; 98.9%) and *eae*_gamma_ (n = 15; 93.8%), respectively. For *ehxA*, subtype C predominated among the big six serogroups and was found in all 16 O145 isolates and most O26 (n = 126; 96.9%), O111 (n = 87; 92.6%), and O121 (n = 46; 93.9%) isolates (Fig. 4C). *ehxA-*F predominated in O45 (n = 185; 97.4%) and O103 (n = 131; 59.6%), though a subset of O103 isolates possessed *ehxA*-C instead. In some cases, serogroups with distinct H antigens possessed different alleles. The O103:H2 isolates, for instance, had either *ehxA* subtypes C or F plus *eae*_epsilon_, while the six O103:H11 isolates had *ehxA*-C and *eae*_beta_. Two of the three O103:H25 isolates had *ehxA*-C and with *eae*_theta_, whereas the the O103:H19 isolate had *ehxA-*F and *eae*_epsilon_.

Indeed, both the gene and allele distributions varied across the phylogeny. Among the 719 isolates with *stx1* only and the 824 with *eae*, 57.0% and 50.5%, respectively, belonged to subclade A in clade I. The same was true for 48.6% of the 839 *ehxA*-positive isolates. Most *stx2* isolates were in subclade C (n = 52; 50.5%) or were singletons (n = 32; 31.1%), which were the most diverse with six *eae* and six *ehxA* alleles represented (Fig. 3). Comparatively, each clade/subclade had 1–2 alleles per gene. Some STs within a subclade had multiple alleles shared by multiple serotypes. The predominant STs 119 and 106 in subclades A and D, respectively, had several *eae* alleles as did ST-157 in subclade B. ST-119 also had isolates with three *ehxA* alleles representing multiple serotypes, while ST-1037 of clade II had two. A subset of lineages had STs with the same combination of *eae* and/or *ehxA* alleles. Subclade G isolates, for instance, had *eae*_beta_ and *ehxA*-F despite having differing serotypes. This widespread distribution of some alleles across the phylogeny is indicative of horizontal gene transfer.

## Discussion

Non-O157 STEC infections have been steadily increasing in frequency in the U.S. since the increased use of culture-independent diagnostic tests^3–5^. Our examination of isolates and case reports in Michigan over an 18-year period indicates that the upward trend is continuing. The diversity of serogroups and serotypes has also increased, thereby highlighting the importance of continued surveillance to monitor disease frequencies and pathogen characteristics. Knowing the limitations associated with surveillance methods, however, is important when evaluating disease trends. The frequency of non-O157 STEC in the earlier time period (2001–2007) in Michigan, for example, likely underestimates the true frequency given that a sentinel surveillance system was utilized^16^. Although the 19-fold increase in non-O157 STEC prevalence observed between the earlier and later (2008–2018) time periods may overestimate the magnitude of the increase, the increasing trend is consistent with national reports^4,5^. Because not all patients with non-O157 STEC infections seek medical care due to differences in access to health care facilities or socioeconomic status^20^, all reported frequencies may be underestimates. The increasing diversity of STEC serogroups over time may also be partly due to the transition from culture-based to culture independent tests, which enhance the likelihood of STEC detection. Despite these limitations, this comprehensive evaluation of STEC was needed to define trends and identify bacterial traits and epidemiological factors linked to infection. Although a large percentage of patient data was missing as is often the case in long-term epidemiological studies^21^, analyzing data from a subset of complete records is useful to inform future studies.

Similar to prior reports^3,4,6,17^, the big six serogroups predominated over the 18-years ranging from 33 to 100% of the total per year with O103 (24.6%) and O45 (21.3%) predominating. The finding that O45:H2 was the only serotype isolated in every year and was the second most common overall is notable given that it was less common at FoodNet sites^3,4,22^. Indeed, geographic variation likely impacts STEC diversity and frequencies as well as clinical outcomes. Variable infection rates have been observed across FoodNet sites^3^ and Michigan differs from other sites in that it is largely an agricultural state with a high density of livestock, particularly cattle, which are important STEC reservoirs. Similar to other studies in different geographic locations^23,24^, we observed a higher proportion of cases in counties with high versus low cattle densities, which may impact serogroup distributions^24^. Our finding that O45:H2 infections were significantly more common in patients residing in counties with greater cattle densities provides additional support for this relationship. By contrast, the significant association between serogroup O103:H2 and low cattle densities suggests a different source for these infections, although more in-depth studies are required to test this hypothesis.

Several serogroups were more common in patients of certain age groups. Older adults and the elderly, for example, had higher frequencies of serogroups outside of the big six. Although international travel history was not known for these cases, older individuals are more likely to travel, which has been shown to enhance the risk of non-O157 infections^3^. Indeed, varying distributions of non-O157 STEC serogroups have been reported in most other countries^25^ and we observed greater STEC diversity in the older cases. Specifically, cases over  18 years had 64 distinct serogroups (79 serotypes) versus 21 serogroups (27 serotypes) for children ≤ 18. Future studies should therefore examine travel status to classify strain types associated with domestic and international travel.

The increased frequency of non-O157 STEC among patients between 11 and 29 years differs from O157 distributions. The latter are typically more common in children ≤ 10 years with a greater risk of HUS developing in those under five^17,26,27^. In Michigan, the average patient age was 29 and is consistent with data from Connecticut^28^, though varying age distributions have been observed elsewhere. Factors responsible for the association with age are not clear. One report indicated that adults between 20 and 39 years more frequently ate out or consumed fast food than adults over 40^29^. Such behavioral differences along with improper handling or cooking of foods could increase exposure risks among young adults. Likewise, prior studies have also observed more infections in females^6,28^ and suggest that age- and sex-specific behaviors could alter the risk and severity of these infections. Supporting data comes from our multivariate analysis showing that females and patients over 65 years were more likely to be hospitalized.

It is also notable that epidemiological associations were identified for specific serogroups (e.g., O45 and O111) but not the lineages (subclades A and D, respectively) associated with those serogroups. Intriguingly, these data suggest that serogroups and serotypes are more important predictors of disease outcomes and risk factors than lineages defined by MLST. Associations between O111 and O45 and hospitalization have been reported previously^6,28^ with O111 being more important for HUS^6^. Isolates representing subclades A and D, which predominated in Michigan and comprised 78.5% of all non-O157 STEC recovered, had unique characteristics. Despite the wide range of serogroups and STs, isolates in these two subclades more frequently possessed *eae* and *ehxA*, two factors linked to enhanced virulence^8,11^, as well as specific subtypes of each gene. Since 10.9% of isolates lacked either gene, however, other bacterial or host factors must also play a role in disease progression in some individuals.

Subclade A isolates all harbored *eae*_epsilon_, while subclade D isolates had *eae*_beta_ and *eae*_theta_. The latter is consistent with a report showing that ST-106, the predominant ST within subclade D, can be differentiated into distinct lineages based on *eae* allele and LEE integration site by MLST^30^ and WGS^31^. Although the three predominant *eae* subtypes identified have been linked to disease elsewhere, the distribution varies by geographic location^11,31–33^. Contrary to two prior studies describing relationships between *eae* presence and certain *ehxA* subtypes^13,14^, four of the 356 (1.1%) *ehxA*-F isolates lacked *eae* and some *ehxA*-A (n = 2 of 35) and D (n = 1 of 3) isolates had *eae*. Despite the correlation between specific *ehxA* subtypes (e.g., A and E) and animal sources^13,14^, many (n = 46; 5.5%) clinical isolates of multiple serogroups possessed these subtypes. Missing epidemiological data prevented an assessment of relationships between animal contact or food consumption history and molecular characteristics. Similar to distributions reported in the U.S.^14^, *ehxA*-C and *ehxA*-F predominated in Michigan with most representing big six serogroups and subclades A or D. Together, these data highlight how variation in horizontally acquired virulence determinants contributes to diversity in non-O157 STEC and that factors in different locations may impact the evolution, distribution and frequency of such elements.

The identification of multiple serogroups and virulence gene profiles in some STs further highlights the diversity and provides additional support for parallel evolution^34,35^. Because of some conflicting relationships between STs in the neighbor-joining phylogeny versus the neighbor-net analysis, it is clear that recombination also plays a role in STEC evolution. One WGS study found that recombination caused conflicting signals in the phylogeny that altered relationships among four STEC isolates^35^. Herein, we have detected evidence for recombination, which has contributed to the emergence of related genotypes and the diversification of novel lineages unique to Michigan. Recombination also likely plays a role in serogroup switching via the exchange of O-antigen genes between serogroups as was suggested for O26, O103 and O111 isolates possessing H11^30,36^. These studies showed that certain H-antigens grouped to specific branches of the phylogeny and comprised multiple serogroups with common ancestors. Our data support these findings for a subset of lineages, but also show that some subclades contained multiple serotypes and H-antigens. Such enhanced diversity within related lineages could be due to the evaluation of a larger sample size, other factors unique to Michigan, or the inability of MLST to differentiate some close relatives. ST-106 within subclade D, for instance, mainly had O111:H8 and O26:H11 isolates but O103:H11, O123:H11, O177:H11, O151:H16, and O118:H16 isolates were also included. The H11 isolates were distinct from the H8 isolates as they possessed *eae*_beta_ and not *eae*_theta_, consistent with data showing that ST-106 comprises separate lineages that can be differentiated by *eae* alleles^31^. Moreover, the ST-106 isolates with H16 had *eae*_beta_ as did all isolates comprising ST-104, a related genotype representing four serogroups with only H11 antigens.

These data highlight the high level of strain variation in non-O157 STEC due to recombination and horizontal gene transfer, which occurs in parallel across locations despite variation in the virulence determinants. Our findings are consistent with WGS and MLST studies^30,31,35,36^, yet future work should involve a more comprehensive genomic analysis to better define evolutionary relationships and virulence traits unique to each lineage. Although the seven MLST loci are highly conserved with no evidence of selection^34^, examining a larger subset of informative and slowly evolving proteins is critical to understand phylogenetic relationships^37^. The latter analyses would be particularly meaningful for those closely related MLST lineages that predominate in Michigan and other geographic locations. Improvements in WGS analyses to characterize non-O157 STEC will continue to improve surveillance methods and outbreak investigations in public health settings, while more comprehensive genomic analyses will further enhance understanding of evolution and diversity across strain populations.

## Methods

### Study population

The Michigan Department of Health and Human Services recovered 894 non-O157 STEC isolates between 2001 and 2018. Patient data were extracted from the web-based MDSS platform (https://www.michigan.gov/mdss). Data collection was performed in accordance with approved ethical standards and authorized by the Institutional Review Boards at MSU (#10-736SM) and the MDHHS (842-PHALAB). All records were anonymized. Epidemiological associations were examined using the Likelihood Ratio Chi-Square (χ^2^) test or Mantel–Haenszel Chi-Square test for trends; sample sizes less than five were evaluated using the Fisher’s exact test in SAS v9.3; p < 0.05 was considered significant. Rural versus urban residence was assigned based on county-level data designated by the National Center for Health Statistics^38^, whereas cattle densities per county were extracted from a 2019 USDA report^39^.

### Whole genome sequencing (WGS) and bioinformatics

Following overnight growth (37 °C) in Luria–Bertani broth, DNA was isolated using the Wizard® Genomic DNA kit for isolates from 2001–2006 and the Qiagen DNAeasy kit for the remainder. Libraries were prepped with the Illumina Nextera XT kit and sequenced on the Illumina MiSeq (2 × 250 reads) as described^19^.

Raw sequencing reads were processed with Trimmomatic to trim adapters and remove sequences with a quality score less than 20 (Q20) or less than 100 nucleotides in length^40^ before quality assessment with FastQC (bioinformatics.babraham.ac.uk/projects/fastqc). De novo assembly was performed with Spades3.10.1 (kmers 21, 33, 55, 77, 99, 127) with error correction to minimize mismatching^41^. All non-O157 STEC genome sequences were deposited in the National Center for Biotechnology Information (NCBI) under BioProjects PRJNA596289, PRJNA514245, PRJNA218110, and PRJNA368991 with each strain having a unique accession number.

Sequences for *stx, wzx/wzy* (O-antigen) and *fliC* (H-antigen) were identified using Abricate (www.github.com/tseemann/abricate) via the Center for Genomic Epidemiology (www.genomicepidemiology.com). Isolates with similar *wzy* and/or *wzx* genes that lacked a complete secondary gene (*wzt* and/or *wzm*) were classified as one of the two serogroups. NT isolates lacked a complete set of genes, thereby preventing the serogroup classification for a subset of isolates. As described^19^, we extracted sequences specific for four *stx1* (a-d) subtypes and seven *stx2* (a-g) subtypes as well as 14 *eae* and six *ehxA* subtypes using published sequences available in the NCBI (Supplementary Table S6). Isolates lacking any of these sequences were classified as negative for the gene, whereas isolates with incomplete sequences were considered NT. Seven housekeeping loci were also extracted for MLST via the Whittam scheme^42^. Sequence types (STs) were assigned with EcMLSTv1.2 (http://www.shigatox.net). For all genes, bioinformatic scripts were used to parse results from a local Basic Local Alignment Search Tool (BLAST)^43^. Sequences specific to each query were extracted from the genomes using an E-value = 0.0001 to ensure a high degree of sequence specificity^44^. MEGAX^45^ aligned MLST sequences with CLUSTALW for construction of a neighbor-joining phylogeny, whereas a neighbor-net phylogeny was constructed using Splitstree4^46^. The PHI was used to evaluate recombination.

## Supplementary Information

Supplementary Information.
