# Supplementary material for: Population structure and genetic diversity of non-O157 Shiga toxin-producing Escherichia coli (STEC) clinical isolates from Michigan
Source: Sci Rep. 2021 Feb 24;11:4461. doi: 10.1038/s41598-021-83775-z (PMC7904848; doi:10.1038/s41598-021-83775-z)

Supplementary Information

Population structure and genetic diversity of non-O157 Shiga toxin-producing *Escherichia coli* (STEC) clinical isolates from Michigan

Heather M. Blankenship[1], Rebekah E. Mosci[1], Stephen Dietrich[2], Elizabeth Burgess[2], Jason Wholehan[2], Karen McWilliams[3], Karen Pietrzen[3], Scott Benko[3], Ted Gatesy[3], James. T. Rudrik[2], Marty Soehnlen[2] and Shannon D. Manning[1*]

[1]Departments of Microbiology and Molecular Genetics, Michigan State University, East Lansing, Michigan, 48824, USA; [2]Michigan Department of Health and Human Services, Bureau of Laboratories, Lansing, Michigan, 48906, USA; [3]Michigan Department of Agriculture and Rural Development, East Lansing, Michigan, 48823, USA

Corresponding author: Shannon D. Manning, Ph.D., Department of Microbiology and Molecular Genetics, Michigan State University, 1129 Farm Lane, East Lansing, Michigan, 48824; mannin71@msu.edu; 517 884-2033; fax: 517 353-8957

Table S1. Differences in the proportion of non-O157 Shiga toxin-producing *Escherichia coli* cases stratified by epidemiological variables.

Case characteristics	No. [a]	(%)	Chi square [b]	DF	P value
Age (years)			246.6	3	<0.0001
0-10	162	(18.5)			
11-29	393	(45.0)			
30-64	241	(27.6)			
≥65	78	(8.9)			
Sex			29.4	1	<0.0001
Male	346	(40.7)			
Female	504	(59.3)			
Time period (years)			726.7	1	<0.0001
2001-2007	44	(4.9)			
2008-2018	850	(95.1)			
Season [c]			153.3	3	<0.0001
Winter	110	(12.4)			
Spring	181	(20.4)			
Summer	362	(40.9)			
Fall	233	(26.3)			
Residence classification by county			7.7	1	0.0055
Rural	298	(56.0)			
Urban	234	(44.0)			
Residence classification by region			502.8	8	<0.0001
Northwest	27	(5.1)			
Northeast	17	(3.2)			
West central	17	(3.2)			
Central	43	(8.1)			
East central	20	(3.8)			
South central	69	(13.0)			
Southwest	121	(22.7)			
Southeast	194	(36.5)			
Upper Peninsula	24	(4.5)			
Cattle density in resident county [d]			36.1	1	<0.0001
Low (<8400 cattle)	157	(35.7)			
High (≥8400 cattle)	283	(64.3)			

Dairy cattle density in resident county [c]			1.3	1	0.26
Low (<3300 cattle)	200	(47.3)			
High (≥3300 cattle)	223	(52.7)			

[a] Number of isolates may not add up to the total number of cases for some variables due to missing data. Limited data was available for cases with isolates submitted after 2015.

[b] Significant differences were identified using the Chi-square Test for Equal Proportions; DF = degree of freedom.

[c] Season was broken down as follows: summer (June-August), fall (September-November), winter (December-February), spring (March-May) based on the collection date, though the stool arrival or onset dates were used for those missing collection dates.

[d] Cattle density was not known for multiple counties with high case counts.

Table S2. Demographic and clinical symptoms associated with hospitalization among patients with non-O157 Shiga toxin-producing *Escherichia coli* infections.

Characteristics	No. of Cases[a]	Hospitalized No.	(%)	OR (95% CI)[b]	P value
**Univariate analysis**					
Age (years)					
0-10	102	14	(13.7)	1.0	-
11-29	234	69	(29.5)	2.6 (1.40, 4.94)	0.0032
30-64	138	47	(34.1)	3.2 (1.67, 6.31)	0.0006
≥65	41	22	(53.7)	7.3 (3.16, 16.75)	<0.0001
Sex					
Male	205	48	(23.4)	1.0	-
Female	301	101	(33.6)	1.7 (1.10, 2.47)	0.013
Season					
Winter, Spring, Fall	306	103	(33.7)	1.0	-
Summer	210	50	(23.8)	0.6 (0.41, 0.92)	0.016
Diarrhea with blood					
No	212	42	(19.8)	1.0	-
Yes	290	109	(37.6)	2.4 (1.61, 3.68)	<0.0001
Abdominal cramps					
No	99	19	(19.2)	1.0	-
Yes	403	132	(32.8)	2.1 (1.19, 3.53)	0.0064
Body aches					
No	395	109	(27.6)	1.0	-
Yes	107	42	(39.3)	1.7 (1.09, 2.65)	0.022
Residence by county					
Rural	289	78	(27.0)	1.0	-
Urban	221	70	(31.7)	1.3 (0.85, 1.84)	0.25
**Multivariate analysis**[c]					
Age ≥65 years				3.8 (1.85, 7.94)	<0.0001
Female				1.8 (1.18, 2.83)	0.0057
Summer				0.6 (0.39, 0.93)	0.0218
Diarrhea with blood				2.4 (1.55, 3.84)	<0.0001

[a] Number of isolates may not add up to the total for some variables due to missing data. Limited data was available for cases with isolates submitted after 2015.

[b] 95% confidence interval for the odds ratio (OR)

[c] Multivariate analysis using forward stepwise logistic regression included variables with p-values >0.2 in the univariate analysis and potential confounders. Variables included: age, sex, residence location, season, and symptoms (diarrhea with blood, abdominal cramping, and body aches). The Homer and Lemeshow Goodness-of-Fit test indicates that the model is supported (p=0.369).

Table S3. Distribution and frequency of 67 non-O157 serotypes among 894 Shiga toxin-producing *Escherichia coli* isolates recovered in Michigan, 2001-2018. A subset of strains was classified as more than one serogroup if they possessed similar *wzy* and/or *wzx* genes but lacked a complete secondary gene (*wzt* and/or *wzm*). NT=non-typeable; *Big six serogroup.

Serotype	Number (%) from 2001-2007	Number (%) from 2008-2018	Total Number (%)
ONT:H16	0 (0.0)	1 (0.1)	1 (0.1)
ONT:H19	0 (0.0)	6 (0.7)	6 (0.7)
ONT:H2	1 (2.3)	0 (0.0)	1 (0.1)
ONT:H25	0 (0.0)	1 (0.1)	1 (0.1)
ONT:H28	0 (0.0)	2 (0.2)	2 (0.2)
ONT:H31	0 (0.0)	1 (0.1)	1 (0.1)
ONT:H32	0 (0.0)	2 (0.2)	2 (0.2)
ONT:H45	1 (2.3)	0 (0.0)	1 (0.1)
ONT:H49	0 (0.0)	1 (0.1)	1 (0.1)
*O103:H11	0 (0.0)	6 (0.7)	6 (0.7)
*O103:H19	0 (0.0)	1 (0.1)	1 (0.1)
*O103:H2	5 (11.4)	205 (24.1)	210 (23.5)
*O103:H25	0 (0.0)	3 (0.4)	3 (0.3)
O109:H10	0 (0.0)	1 (0.1)	1 (0.1)
O110:H28	1 (2.3)	0 (0.0)	1 (0.1)
*O111:H11	0 (0.0)	1 (0.1)	1 (0.1)
*O111:H8	3 (6.8)	90 (10.6)	93 (10.4)
O113:H21	1 (2.3)	1 (0.1)	2 (0.2)
O115:H10	0 (0.0)	1 (0.1)	1 (0.1)
O116:H49	0 (0.0)	1 (0.1)	1 (0.1)
O117:H7	0 (0.0)	1 (0.1)	1 (0.1)
O118:H16	0 (0.0)	1 (0.1)	1 (0.1)
*O121:H19	0 (0.0)	48 (5.7)	48 (5.4)
*O121:H7	0 (0.0)	1 (0.1)	1 (0.1)
O123:H11	0 (0.0)	4 (0.5)	4 (0.5)
O123:H2	0 (0.0)	17 (2.0)	17 (2.0)
O130:H11	0 (0.0)	3 (0.4)	3 (0.3)
*O145:H28	3 (6.8)	13 (1.5)	16 (1.8)
O151:H16	0 (0.0)	11 (1.3)	11 (1.2)
O151:H2	0 (0.0)	5 (0.6)	5 (0.6)
O153/O178:H19	0 (0.0)	2 (0.2)	2 (0.2)
O156:H25	0 (0.0)	4 (0.5)	4 (0.5)
O163:H19	0 (0.0)	1 (0.1)	1 (0.1)
O165:H25	0 (0.0)	5 (0.6)	5 (0.6)
O166:H15	0 (0.0)	1 (0.1)	1 (0.1)
O17/O77:H45	0 (0.0)	1 (0.1)	1 (0.1)

O17:H18	0	(0.0)	1	(0.1)	1	(0.1)
O17:H41	0	(0.0)	2	(0.2)	2	(0.2)
O17:H45	0	(0.0)	2	(0.2)	2	(0.2)
O172:H45	0	(0.0)	1	(0.1)	1	(0.1)
O174:H21	0	(0.0)	1	(0.1)	1	(0.1)
O177:H11	0	(0.0)	1	(0.1)	1	(0.1)
O177:H25	0	(0.0)	3	(0.4)	3	(0.3)
O177:H45	0	(0.0)	1	(0.1)	1	(0.1)
O183:H18	0	(0.0)	3	(0.4)	3	(0.3)
O185:H28	0	(0.0)	1	(0.1)	1	(0.1)
O187:H52	1	(2.3)	0	(0.0)	1	(0.1)
O2:H27	0	(0.0)	1	(0.1)	1	(0.1)
O22:H8	1	(2.3)	1	(0.1)	2	(0.2)
*O26:HNT	0	(0.0)	2	(0.2)	2	(0.2)
*O26:H11	5	(11.4)	123	(14.5)	128	(14.3)
O28ac/O42:H25	0	(0.0)	3	(0.4)	3	(0.3)
O28ac:H25	0	(0.0)	2	(0.2)	2	(0.2)
O38:H21	0	(0.0)	1	(0.1)	1	(0.1)
O44:HNT	0	(0.0)	2	(0.2)	2	(0.2)
*O45:HNT	0	(0.0)	1	(0.1)	1	(0.1)
*O45:H2	17	(38.6)	172	(20.2)	189	(21.1)
O49:HNT	0	(0.0)	1	(0.1)	1	(0.1)
O49:H16	1	(2.3)	0	(0.0)	1	(0.1)
O5:H9	1	(2.3)	29	(3.4)	30	(3.4)
O55:H12	0	(0.0)	1	(0.1)	1	(0.1)
O55:H7	0	(0.0)	2	(0.2)	2	(0.2)
O69:H11	1	(2.3)	6	(0.7)	7	(0.8)
O7:H7	0	(0.0)	1	(0.1)	1	(0.1)
O71:H11	0	(0.0)	24	(2.8)	24	(2.7)
O713/O17/O77:H45	0	(0.0)	2	(0.2)	2	(0.2)
O76:H19	1	(2.3)	0	(0.0)	1	(0.1)
O8:H17	0	(0.0)	1	(0.1)	1	(0.1)
O8:H19	0	(0.0)	3	(0.4)	3	(0.3)
O80:H2	0	(0.0)	2	(0.2)	2	(0.2)
O84:H2	0	(0.0)	4	(0.5)	4	(0.5)
O85:H1	0	(0.0)	1	(0.1)	1	(0.1)
O88:H25	0	(0.0)	2	(0.2)	2	(0.2)
O91:H14	1	(2.3)	3	(0.4)	4	(0.5)
O91:H21	0	(0.0)	1	(0.1)	1	(0.1)
O98:H21	0	(0.0)	1	(0.1)	1	(0.1)
**Total**	44		850		894	

Table S4. Serogroup distribution of non-O157 Shiga toxin-producing *Escherichia coli* isolates and epidemiological associations.

Characteristics	O26 (n=130)		O45 (n=190)		O103 (n=220)		O111 (n=94)		O121 (n=49)		O145 (n=16)		Others (n=195)	
	No.	(%)	No.	(%)	No.	(%)	No.	(%)	No.	(%)	No.	(%)	No.	(%)
Age (years)														
0-10 (n=162)	29	(17.9)	29	(17.9)	38	(23.5)	22	(13.6)	10	(6.2)	3	(1.9)	31	(19.1)
11-29 (n=393)	68	(17.3)	97	(24.7)	96	(24.4)	36	(9.2)	22	(5.6)	4	(1.0)	70	(17.8)
30-64 (n=241)	22	(9.1)	51	(21.2)	68	(28.2)	24	(10.0)	12	(5.0)	6	(2.5)	58	(24.1)
≥65 (n=78)	7	(9.0)	12	(15.4)	18	(23.1)	10	(12.8)	5	(6.4)	3	(3.9)	23	(29.5)
Sex														
Male (n=346)	54	(15.6)	82	(23.7)	89	(25.7)	40	(11.6)	17	(4.9)	6	(1.7)	58	(16.8)
Female (n=504)	72	(14.3)	103	(20.4)	122	(24.2)	48	(9.5)	32	(6.4)	9	(1.8)	118	(23.4)
Residence														
Rural (n=298)	48	(16.1)	79	(15.4)	72	(24.2)	29	(9.7)	17	(5.7)	7	(2.4)	46	(15.4)
Urban (n=234)	39	(16.7)	62	(26.5)	57	(24.4)	25	(10.7)	12	(5.1)	3	(1.3)	36	(15.4)
Season[a]														
Winter (n=110)	14	(12.7)	32	(29.1)	26	(23.6)	6	(5.5)	5	(4.6)	1	(0.9)	26	(23.6)
Spring (n=181)	26	(14.4)	27	(14.9)	43	(23.8)	16	(8.8)	8	(4.4)	5	(2.8)	56	(30.9)
Summer (n=362)	59	(16.3)	76	(21.0)	95	(26.2)	42	(11.6)	23	(6.4)	6	(1.7)	61	(16.9)
Fall (n=233)	29	(12.5)	54	(23.2)	56	(24.0)	28	(12.0)	13	(5.6)	4	(1.7)	49	(21.0)
Time period (years)														
2001-2007 (n=44)	5	(11.4)	17	(38.6)	5	(11.4)	3	(6.8)	0	(0.0)	3	(6.8)	11	(25.0)
2008-2018 (n=850)	125	(14.7)	173	(20.4)	215	(25.3)	91	(10.7)	49	(5.8)	13	(1.5)	184	(21.7)

Cattle density in resident county							
Low (<8400) (n=157)	29 (18.5)	29 (18.5)	56 (35.7)	11 (7.0)	13 (8.3)	4 (2.6)	15 (9.6)
High (≥8400) (n=283)	41 (14.5)	98 (34.6)	57 (20.1)	27 (9.5)	10 (3.5)	4 (1.4)	46 (16.3)
**Clinical Outcomes**							
Hospitalized							
No (n=363)	67 (18.5)	81 (22.3)	101 (27.8)	33 (9.1)	17 (4.7)	5 (1.4)	59 (16.3)
Yes (n=153)	17 (11.1)	52 (34.0)	24 (15.7)	19 (12.4)	11 (7.2)	5 (3.3)	25 (16.3)
Diarrhea with blood							
No (n=212)	37 (17.5)	46 (21.7)	58 (27.4)	15 (7.1)	9 (4.3)	1 (0.5)	46 (21.7)
Yes (n=293)	44 (15.0)	87 (29.7)	62 (21.2)	38 (13.0)	17 (5.8)	8 (2.7)	37 (12.6)
Abdominal cramps							
No (n=99)	19 (19.2)	25 (25.3)	29 (29.3)	4 (4.0)	5 (5.1)	0 (0.0)	17 (17.2)
Yes (n=405)	62 (15.3)	108 (26.7)	90 (22.2)	49 (12.1)	21 (5.2)	9 (2.2)	66 (16.3)
Body aches							
No (n=397)	64 (16.1)	101 (25.4)	98 (24.7)	46 (11.6)	20 (5.0)	6 (1.5)	62 (15.6)
Yes (n=107)	17 (15.9)	32 (29.9)	21 (19.6)	7 (6.5)	6 (5.6)	3 (2.8)	21 (19.6)

Note: Percentages are calculated using the characteristic numbers as the denominators, though the number of isolates may not add up to the total for some variables due to missing data. The serogroup genes were missing from 8 isolates and 16 isolates in the "Others" category were classified as non-typeable (NT).

Table S5. Characteristics of non-O157 Shiga toxin-producing *Escherichia coli* cases stratified by lineage defined in the multilocus sequence typing analysis.

Characteristics	Subclade A (n=418) No. (%)	Subclade D (n=271) No. (%)	Subclades B,C,E, F, G (n=112) No. (%)	Clades II/III (n=15) No. (%)	Singletons (n=54) No. (%)
Age (years)					
0-10 (n=160)	68 (42.5)	65 (40.6)	20 (12.5)	2 (1.3)	5 (3.1)
11-29 (n=392)	199 (50.8)	130 (33.2)	44 (11.2)	2 (0.5)	17 (4.3)
30-64 (n=240)	120 (50.0)	57 (23.8)	36 (15.0)	8 (3.3)	19 (7.9)
≥65 (n=78)	31 (39.7)	19 (24.4)	12 (15.4)	3 (3.9)	13 (16.7)
Sex					
Male (n=345)	173 (50.1)	112 (32.5)	43 (12.5)	4 (1.2)	13 (3.8)
Female (n=501)	232 (46.3)	153 (30.5)	68 (13.6)	10 (2.0)	38 (7.6)
Residence					
Rural (n=294)	154 (52.4)	89 (30.3)	27 (9.2)	3 (1.0)	21 (7.1)
Urban (n=234)	115 (49.2)	78 (33.3)	27 (11.5)	3 (1.3)	11 (4.7)
Season					
Winter (n=110)	59 (53.6)	31 (28.2)	12 (19.9)	1 (0.9)	7 (6.4)
Spring (n=181)	77 (42.5)	59 (32.6)	24 (13.3)	5 (2.8)	16 (8.8)
Summer (n=358)	172 (48.0)	112 (31.3)	49 (13.7)	6 (1.7)	19 (5.3)
Fall (n=232)	110 (47.4)	73 (31.5)	32 (13.8)	3 (1.3)	14 (6.0)
Time period (years)*					
2001-2007 (n=44)	21 (47.7)	9 (20.5)	3 (6.8)	1 (2.3)	10 (22.7)
2008-2018 (n=845)	398 (47.1)	270 (32.0)	116 (13.7)	15 (1.8)	46 (5.4)

Cattle density in resident county					
Low (<8400 cattle) (n=157)	85 (54.1)	42 (26.8)	18 (11.5)	3 (1.9)	9 (5.7)
High (≥8400 cattle) (n=279)	156 (55.9)	87 (31.2)	21 (7.5)	1 (0.4)	14 (5.0)
**Clinical Outcomes**					
Hospitalized					
No (n=360)	181 (50.3)	120 (33.3)	34 (9.4)	6 (1.7)	19 (5.3)
Yes (n=153)	76 (49.7)	43 (28.1)	19 (12.4)	0 (0.0)	15 (9.8)
Diarrhea with blood					
No (n=211)	107 (50.7)	61 (28.9)	23 (10.9)	2 (1.0)	18 (8.5)
Yes (n=291)	145 (49.8)	99 (34.0)	28 (9.6)	4 (1.4)	15 (5.2)
Abdominal cramps					
No (n=98)	53 (54.1)	27 (27.6)	11 (11.2)	1 (1.0)	6 (6.1)
Yes (n=403)	198 (49.1)	133 (33.0)	40 (9.9)	5 (1.2)	27 (6.7)
Body aches					
No (n=395)	199 (50.4)	133 (33.7)	38 (9.6)	3 (0.8)	22 (5.6)
Yes (n=106)	52 (49.1)	27 (25.5)	13 (12.3)	3 (2.8)	11 (10.4)
**Molecular characteristics**					
Shiga toxin gene profile*					
stx1 (n=719)	410 (57.0)	248 (34.5)	43 (6.0)	6 (0.8)	12 (1.7)
stx2 (n=103)	1 (0.1)	3 (2.9)	62 (60.2)	5 (4.9)	32 (31.1)
stx1, *stx2* (n=67)	8 (11.9)	28 (41.8)	14 (20.9)	5 (7.5)	12 (17.9)
Presence of *eae**					
No (n=65)	3 (4.6)	2 (3.1)	21 (32.3)	14 (21.5)	25 (38.5)
Yes (n=824)	416 (50.5)	277 (33.6)	98 (11.9)	2 (0.2)	31 (3.8)

Presence of *ehxA**					
No (n=50)	11 (22.0)	13 (26.0)	9 (18.0)	6 (12.0)	11 (22.0)
Yes (n=839)	408 (48.6)	266 (31.7)	110 (13.1)	10 (1.2)	45 (5.4)
Serogroup*					
Others (n=193)	22 (11.4)	50 (25.9)	68 (35.2)	15 (7.8)	38 (19.7)
Big six (n=696)	397 (57.0)	229 (32.9)	51 (7.3)	1 (0.1)	18 (2.6)
Predominant big six serotypes*					
All others (n=177)	59 (33.3)	22 (12.4)	50 (28.3)	14 (7.9)	32 (18.1)
O26:H11 (n=128)	0 (0.0)	0 (0.0)	128 (100.0)	0 (0.0)	0 (0.0)
O26:HNT (n=1)	0 (0.0)	1 (100.0)	0 (0.0)	0 (0.0)	0 (0.0)
O45:H2 (n=188)	188 (100.0)	0 (0.0)	0 (0.0)	0 (0.0)	0 (0.0)
O103:H2 (n=210)	209 (99.5)	0 (0.0)	0 (0.0)	1 (0.5)	0 (0.0)
O103:H11 (n=6)	0 (0.0)	6 (100.0)	0 (0.0)	0 (0.0)	0 (0.0)
O103:H19 (n=1)	0 (0.0)	0 (0.0)	0 (0.0)	0 (0.0)	1 (100.0)
O103:H25 (n=3)	0 (0.0)	0 (0.0)	3 (100.0)	0 (0.0)	0 (0.0)
O111:H8 (n=93)	0 (0.0)	93 (100.0)	0 (0.0)	0 (0.0)	0 (0.0)
O111:H11 (n=1)	0 (0.0)	1 (100.0)	0 (0.0)	0 (0.0)	0 (0.0)
O121:H7 (n=1)	0 (0.0)	0 (0.0)	0 (0.0)	0 (0.0)	1 (100.0)
O121:H19 (n=48)	0 (0.0)	0 (0.0)	48 (100.0)	0 (0.0)	0 (0.0)
O145:H28 (n=16)	0 (0.0)	0 (0.0)	0 (0.0)	0 (0.0)	16 (100.0)

Note: Percentages are calculated using the characteristic numbers as the denominators, though the number of isolates may not add up to the total for some variables due to missing data.

*indicates significantly different distributions using the Mantel Haenszel Chi Square test $p \leq 0.05$

Table S6. Accession numbers linked to each gene variant analyzed in the study. Sequences and analytical methods for extracting these gene alleles were originally published in 2020 in Blankenship et al. (Frontiers Microbiol.11:59).

Gene	Allele	National Center for Biotechnology Information (NCBI) Accession number(s)
**Shiga toxin (*stx*)**	*stx1a*	M19473.1, AM230662.1
	stx1c	Z36901.1, AB048237.1
	stx1d	AY170851.1
	stx2a	X07865.1, EF441609.1
	stx2b	AF043627.1, AB048226.1
	stx2c	M59432.1, EU086525.1
	stx2d	FM998855.1, DQ059012.1
	stx2e	X81418.1, AY332411.1
	stx2f	AJ010730.1, AB472687.1
	stx2g	AY286000.1, AB048227.1
**Entero-hemolysin (*ehx*)**	*ehxA-A*	AY258503.2
	ehxA-B	AP018692.1
	ehxA-C	AP010954.1
	ehxA-D	EF204927.1
	ehxA-E	EF204923.1
	ehxA-F	AP010959.1
**Intimin (*eae*)**	alpha	AF022236.1, AF530555.1
	beta	MK761162.1, AJ715407.1
	delta	AJ875027.1
	kappa	AJ308552.1
	gamma	CP034384.1
	theta	AF449418.1
	epsilon	AF116899.1, DQ523614.1
	zeta	AJ271407.1
	eta	AJ308550.1, AJ876652.1
	jota	DQ523601.1, AF530553.1
	lambda	AJ715409.1
	mu	AJ705049.1
	nu	AJ705050.1
	xi	AJ705051.1

Figure S1. Total number of non-O157 Shiga toxin-producing *Escherichia coli* isolates that were recovered for whole genome sequencing (WGS) compared to the total number of non-O157 STEC cases reported by the Michigan Department of Health and Human Services (MDHHS) over the 18-year period.

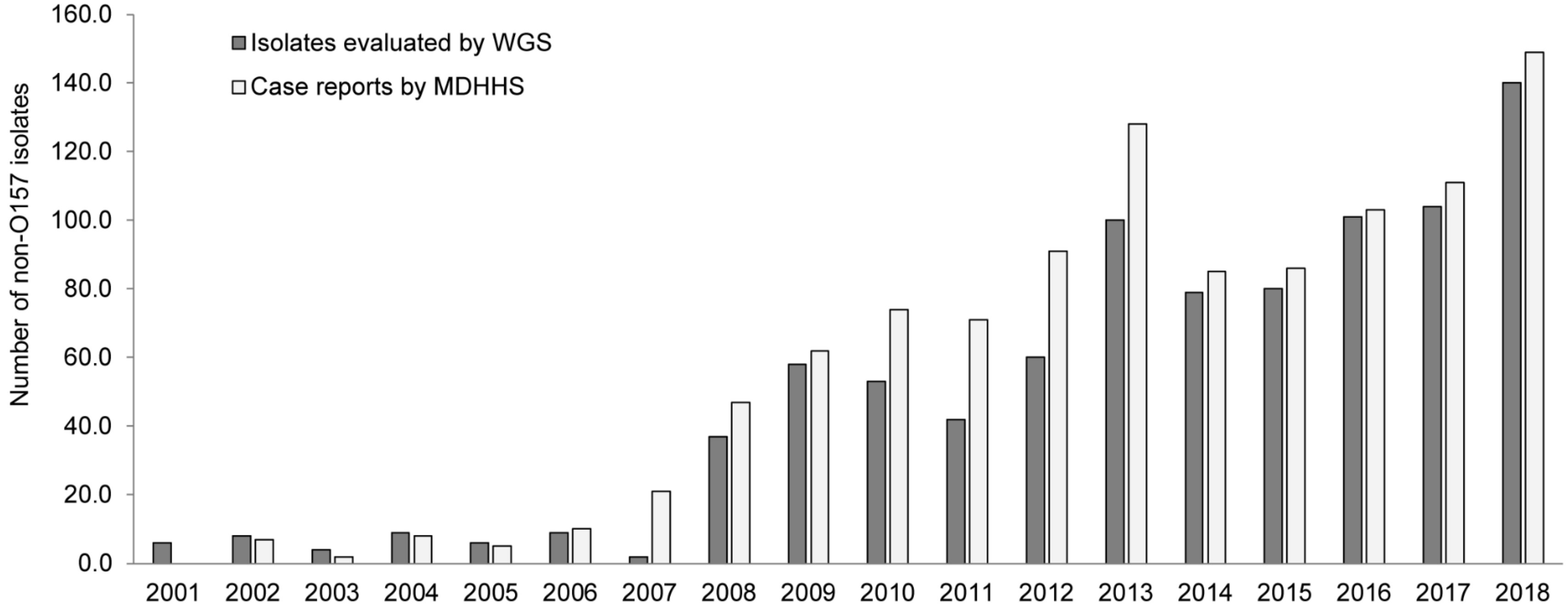

Figure S2. Distribution of age groups among non-O157 Shiga toxin-producing *Escherichia coli* cases in Michigan 2001-2018.

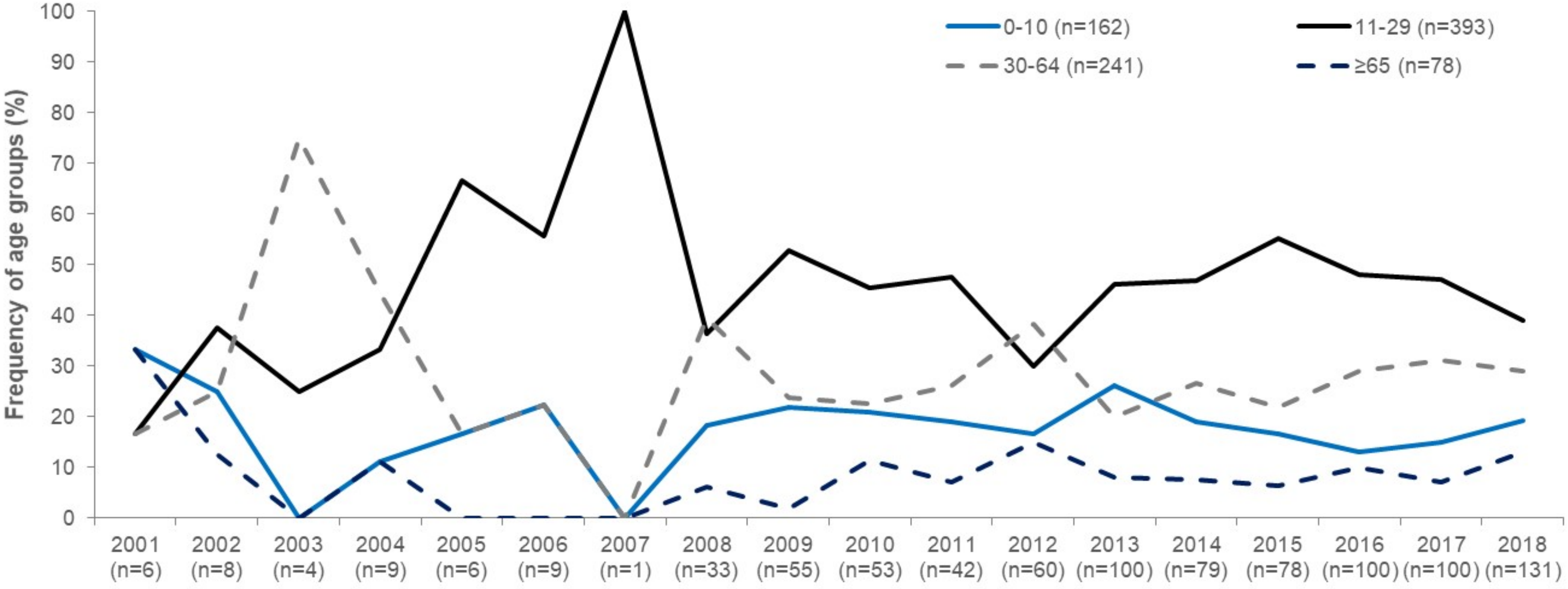

Figure S3. Percentage of non-O157 Shiga toxin-producing *Escherichia coli* representing the top six predominant serogroups, O26, O45, O103, O111, O121, and O145, recovered from Michigan cases, 2001-2018.

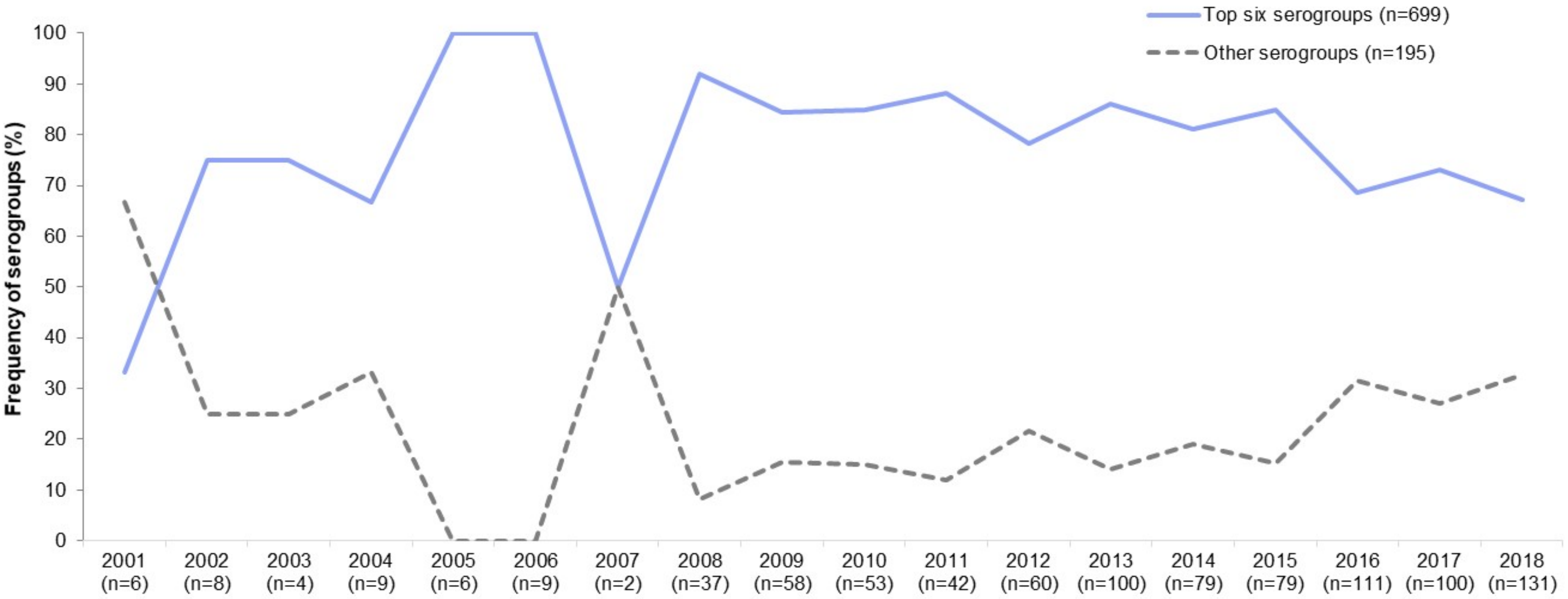

Figure S4. Percentage of the 889 non-O157 Shiga toxin-producing *Escherichia coli* isolates belonging to subclades A and D over an 18-year period in Michigan.

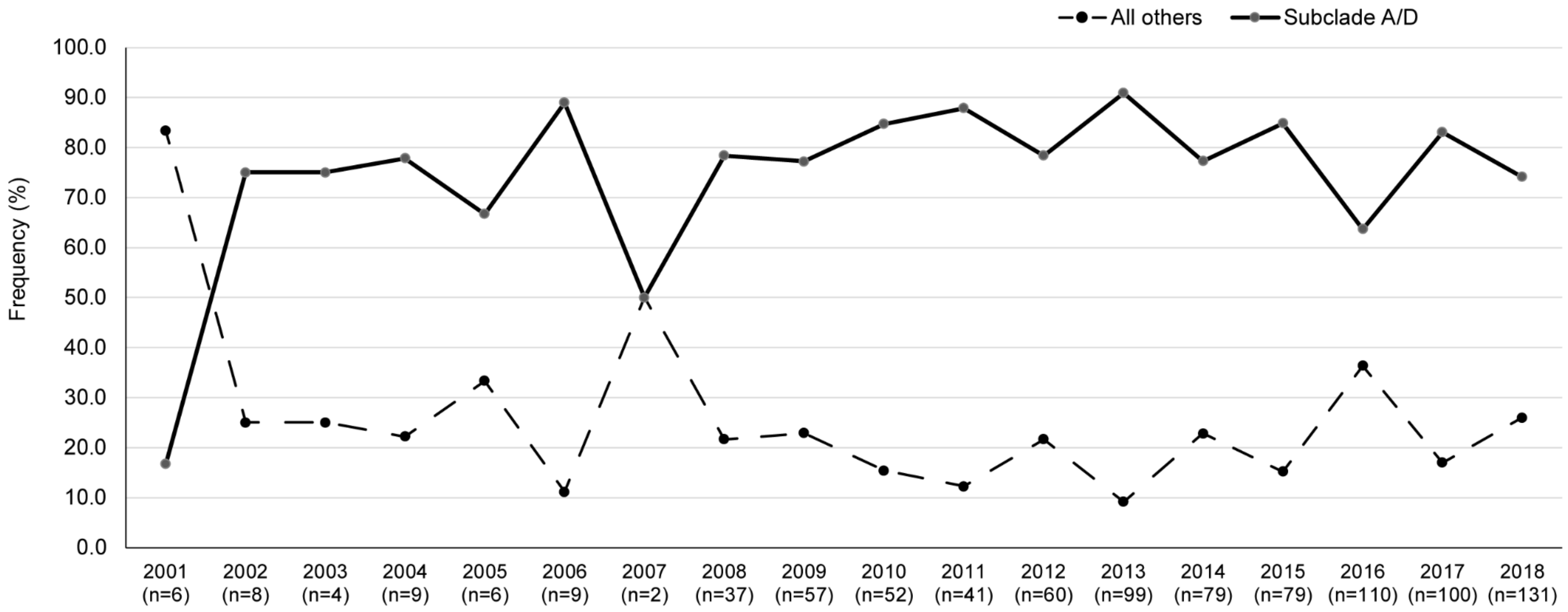

Figure S5. Distribution and frequency of H-antigens by subclade and clade. NT=non-typeable; S=singleton lineage not associated with a clade or subclade in the multilocus sequence typing phylogeny.

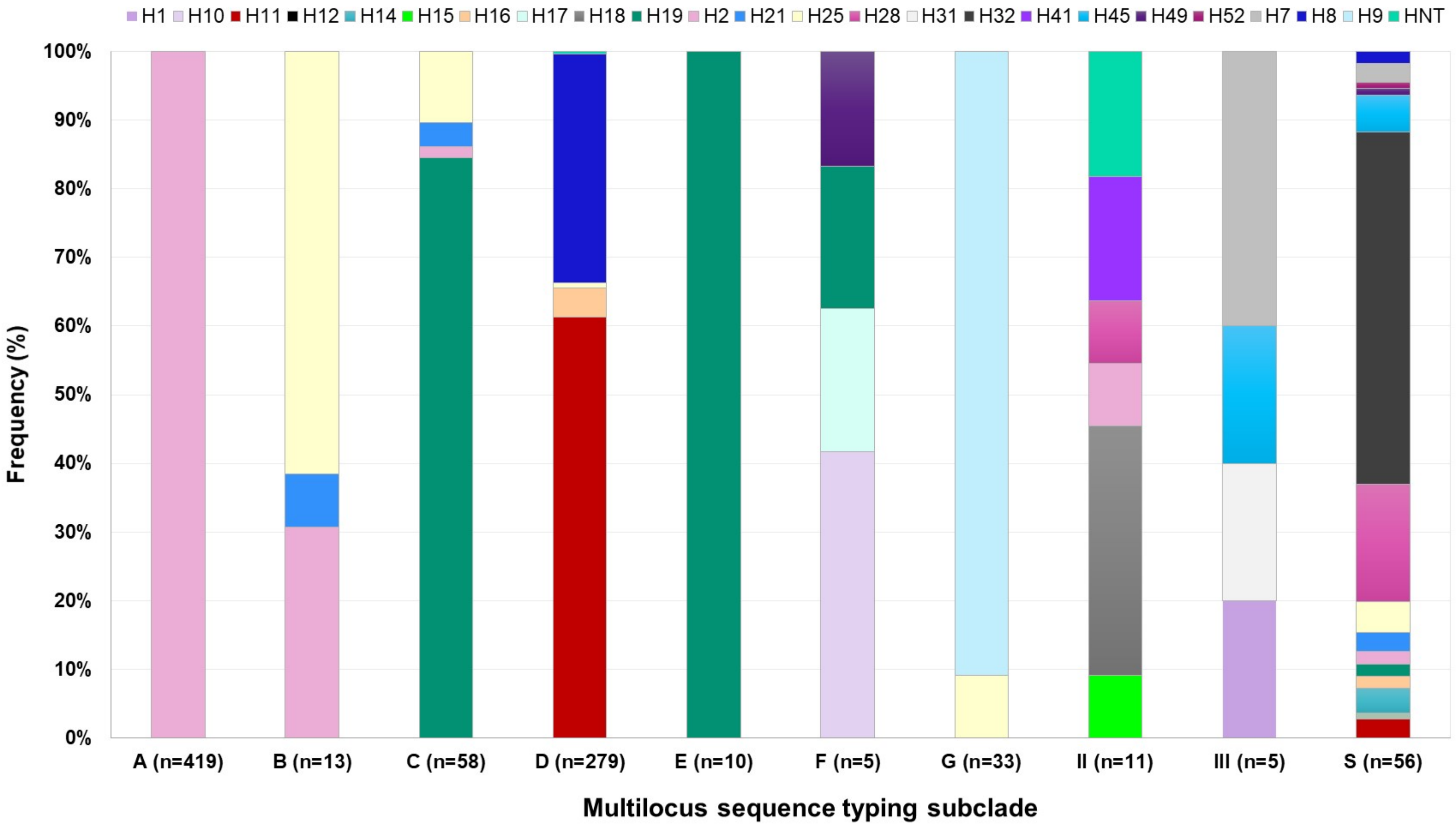

Supplement: Supplementary file 1 — Supplementary Information. [file 41598_2021_83775_MOESM1_ESM.pdf]
